# 360. Evaluation of Cycle Threshold Values in Patients with Symptomatic COVID-19 Infection

**DOI:** 10.1093/ofid/ofab466.561

**Published:** 2021-12-04

**Authors:** Danielle Dixon, Julieta Madrid-Morales, Jose Cadena-Zuluaga, Christopher R Frei

**Affiliations:** 1 University of Texas Health, San Antonio, San Antonio, Texas; 2 University of Texas Health Science Center at San Antonio, Texas, USA, San Antonio, Texas; 3 University of Texas health and science center San Antonio, Audie L. Murphy VA Medical Center, San Antonio, Texas; 4 University of Texas at Austin College of Pharmacy/UT Health San Antonio, San Antonio, Texas

## Abstract

**Background:**

One of the tests used to identify COVID-19 infections is the severe acute respiratory syndrome coronavirus 2 (SARS-CoV-2) reverse transcriptase quantitative polymerase chain reaction (RT-qPCR) test. There is a measure known as the cycle threshold (Ct) value, which provides an indirect measure of viral load. It has been proposed that the Ct value could help with clinical decisions regarding duration of isolation. We hypothesize that Ct values will correlate with symptom duration in a population of veterans with COVID-19 infection.

**Methods:**

We reviewed the records of patients presenting to the emergency department (ED) or admitted to Audie L. Murphy VA Medical Center in San Antonio, Texas with positive SARS-CoV-2 PCR tests. We looked at patients who received multiple SARS-CoV-2 RT-qPCR tests. We compared date of onset of symptoms and cycle threshold values from their initial test to another test ordered after 7, 10, and 20 days from symptom onset. We recorded the Ct value for the N2 and E genes. Patients were classified into mild, severe and critical based on Center for Disease Control and Prevention (CDC) criteria. A Ct value of >30 as threshold for transmissible disease was used based on previously published studies.

**Results:**

We identified 49 patients with more than two SARS-CoV-2 RT-qPCR tests. Patients with mild disease with tests less than or equal to ten days from symptom onset (n=10) had a mean Ct value 23.2 (±5.6) and 26.0 (±5.8) for the E and N2 genes. Patients with mild disease with tests greater than ten days from symptom onset (n=4) had mean Ct values of 26.0 (±6.5) and 27.8 (±6.8). When we stratified the patient population by disease severity, patients with severe and critical disease with tests less than ten days from symptom onset (n=24) had mean Ct values of 20.1 (±7.3) and 23.4 (±7.5). Patients with severe and critical disease greater than twenty days (n=6) had Ct values of 29.0 (±5.1) and 31.1 (±5.4).

**Conclusion:**

We found that Ct values increased with longer symptom duration. We currently use the CDC criteria to discontinue isolation at ten days for mild disease and twenty days for severe and critical disease. The findings of this study suggest that our current practice for duration of isolation correlates with increasing Ct values near or above the threshold for transmissible disease.

**Disclosures:**

**All Authors**: No reported disclosures

